# The relationship between occupational stress, job burnout and quality of life among surgical nurses in Xinjiang, China

**DOI:** 10.1186/s12912-021-00703-2

**Published:** 2021-09-27

**Authors:** Xue Li, Ting Jiang, Jian Sun, Lingyun Shi, Jiwen Liu

**Affiliations:** 1 Department of Public Health, Xinjiang Medical University, 830011 Urumqi, China; 2 Department of Gastroenterology, The Sixth Affiliated Hospital of Xinjiang Medical University, 830000 Urumqi, China; 3 Department of Joint Surgery, the First Affiliated Hospital of Xinjiang Medical University, 830054 Urumqi, China

**Keywords:** Job Burnout, Occupational Stress, Quality of Life, Structural Equation

## Abstract

**Background:**

Nursing is a high-risk occupation that involves exposure to stress. The physical and mental health of nurses is directly related to the quality of medical services, so the quality of life of nurses cannot be ignored. This study is a Chinese nursing study that investigated occupational stress, job burnout, and quality of life of surgical nurses in Xinjiang, China.

**Methods:**

This study employed the cluster random sampling method and carried out a questionnaire survey among 488 surgical nurses from five hospitals from May 2019 to September 2019. The study analyzed the relationship between occupational stress, job burnout and quality of life. The Effort-Reward Imbalance questionnaire (ERI), Maslach Burnout Inventory General Survey (MBI-GS) and the 36-item Short Form Health Survey (SF-36) were used to evaluate occupational stress, job burnout and quality of life among surgical nurses.

**Results:**

A total of 550 questionnaires were distributed in this study, and 488 were retrieved, with an effective recovery rate of 88.73 %. The results revealed that the quality of life score among surgical nurses was not high, and differences were observed in the quality of life score of patients according to gender, age, title, and frequency of night shifts (*P* < 0.05). There was a positive correlation between occupational stress and job burnout. Higher levels of occupational stress and job burnout were associated with a poorer quality of life score. Occupational stress and job burnout were identified as risk factors for quality of life, and the interaction between high levels of stress and burnout seriously reduced quality of life. The structural equation model revealed that occupational stress and job burnout had a direct impact on quality of life, occupational stress had a direct impact on job burnout, and job burnout was identified as a mediating factor in the relationship between occupational stress and quality of life.

**Conclusions:**

Surgical nurses have a high level of occupational stress and burnout, and low quality of life score. Quality of life is correlated with occupational stress and job burnout. According to the individual characteristics and psychological state of nurses, managers can implement personalized intervention measures promptly and effectively to relieve their tension and burnout, and improve the quality of life of surgical nurses.

## Background

The workload is heavy, the working hours are long, and the nursing profession is associated with a high degree of pressure and risk. Chronic and excessive stress can pose serious threats to physical and mental health [1]. Stress not only affects the physical and mental health of medical staff, but also affects their work quality and efficiency [2]. As a special professional group, nurses shoulder the important task of saving lives and promoting health. Their working status and working ability can have a direct impact on the lives and safety of patients [3]. Therefore, the health of nurses should also be highly valued. Research shows that the nursing profession is highly pressurized, the work is intense, and nurses are part of the group that is typically at risk of occupational illness and ill health [4]. Nurses suffer from severe occupational stress and job burnout [5].

Occupational stress refers to the physiological and psychological pressure caused by an imbalance between objective requirements and the adaptability of individuals in the process of work, which is a kind of non-specific abnormal psychological reaction [6]. An appropriate amount of pressure is conducive to improving the work efficiency of workers. However, when the professional knowledge, individual ability, vocational skills and work experience of the workers are not adapted to work-related needs, and when employees are unable to make changes through their own efforts, serious occupational psychological pressure can occur, leading to occupational stress reactions [7]. Occupational stress has the following characteristics: (1) it is persistent; (2) it is difficult to adapt to, often causing emotional fluctuations; (3) it is difficult to change with the requirements of the working environment; (4) it not only affects the employee’s work, but also their personal life outside the workplace; and (5) poor working conditions will eventually harm health [8]. Studies have found that excessive occupational stress may cause hypertension, cardiovascular diseases, digestive system diseases, and joint and muscle diseases, among others, which seriously affect the work efficiency and attendance rate of nursing staff, leading to a lack of human resources [9]. Wei et al. [10] analyzed occupational stress among nursing staff at a tertiary hospital in Xinjiang, and the results showed that the proportion of low occupational stress was 6.1 %, the proportion of medium occupational stress was 47.1 %, and the proportion of high occupational stress was 46.8 %, all of which were higher than the national standard. The long-term alleviation of tension is problematic, and as tension accumulates, nurses will suffer from job burnout [11].

Job burnout refers to the physical, emotional and psychological states of the individual that lead to work-related stress reactions, and it mainly manifests in the form of emotional exhaustion, depersonalization and diminished personal accomplishment[12]. The nursing profession is associated with a high incidence of job burnout [13]. The occupational characteristics of nurses are such that they are faced with many sources of stress. This kind of high-stress work environment can easily cause nurses to experience fatigue [14, 15]. Job burnout will directly reduce the service quality of nurses [16]. Studies have shown that non-burnout nursing staff are significantly less likely to make errors at work than burnout nursing staff, and burnout directly affects the quality of nursing services [17]. Ayman et al. argued that increasing the workload would result in increased working pressures among nurses, leading to exhaustion and occupational stress. Stress and burnout can have a detrimental effect on an organization’s productivity, and can cause serious health and safety hazards in the workplace [18]. According to previous studies, the existence of occupational stress may not necessarily lead to job burnout among nurses, but if stress is experienced over a long period of time and cannot be alleviated effectively, it will lead to job burnout and will further affect quality of life [19].

Quality of life refers to general well-being of individuals with regard to their life goals, expectations, standards, and living conditions, and it is related to the things that they care about. It affects the physiological, psychological, and social functioning of the individual [20]. There are many factors that affect quality of life, such as age, employment mode, professional title, educational level, monthly income, difficulty in balancing work and family, social support, etc. [21, 22]. Nowrouzi et al. found that a good hospital management culture can improve the professional quality of life of nursing staff and reduce their turnover rate [23]. Good or poor quality of life can indirectly and directly affect the work efficiency, work quality, organizational commitment, job satisfaction and even turnover intention of nurses [24, 25]. For nurses, in addition to the above factors that affect their quality of life, negative emotions in daily life and work-related stress can affect their quality of life. Studies have found that job burnout not only affects the physical and mental health of nursing staff, but also reduces their quality of life [26].

A surgical nurse is a nurse who provides holistic care to surgical patients. The service objects that are under the care of surgical nurses are quite special, and they are usually patients who have undergone surgery and trauma. Moreover, the development of diseases is changeable, so nurses are required to have a greater capacity to bear the strains that may be encountered. Due to the continuity, inheritance and service of nursing work, the nursing staff in the ward must provide nursing services to patients 24 h a day without interruption. Therefore, the working hours of surgical nurses generally involve three shifts (day shift; usually from 8 to 4 p.m., swing shift; usually from 4 to 12 p.m., and the graveyard shift; usually from 12 to 8 a.m., and each shift is eight hours in duration. Usually, four or five nurses work the day shift, and they have one hour for their lunch break, due to the large number of patients needing nursing care. In general, two or three nurses work the swing shift and the graveyard shift, and they have no fixed break times. The high work intensity and heavy tasks involved in the work of surgical nurses mean that they are more prone to occupational stress and burnout. Surgical nurses play an important role in the process of patient rehabilitation. The psychological and physiological status of nurses can directly affect their working ability, which means that they may not be able to provide the best quality of service for their patients. They may be more prone to errors as they carry out their nursing duties, which can harm the health of patients. In recent years, studies on the quality of life of nurses mainly focused on the influence of age, gender, marital status, length of service, working years, shifts, job satisfaction, fatigue and other factors related to quality of life [27–30], while there are few studies that have used scales to objectively measure the level of nurses’ occupational stress and burnout. The study examined [31] high levels of stress and burnout among nurses, but few studies have analyzed the relationship between these two factors and quality of life. Clarifying the relationship between these three factors can provide a referential framework for managers to help them to formulate effective intervention measures for nurses who have different physical and mental health states. This study investigated occupational stress, job burnout and the quality of life of surgical nurses from five hospitals in Xinjiang, China, using a questionnaire to analyze the impact of occupational stress and job burnout on the quality of life of nurses. By developing a structural equation model, this study further analyzed the relationship between these three factors.

## Methods

### Study population

This study employed a cluster random sampling method, and the survey was carried out from May 2019 to September 2019. Five affiliated hospitals of Xinjiang Medical University (the First Affiliated Hospital, the Third Affiliated Hospital, the Fourth Affiliated Hospital, the Fifth Affiliated Hospital, and the Sixth Affiliated hospital) were selected, and 10 nurses were randomly selected from the surgical department of each hospital. All five hospitals were tertiary hospitals, with comparable medical and health service capacity. (Tertiary hospitals are medical institutions classified in accordance with the current Administrative Measures on Hospital Classification in China. Hospitals are graded on a scale of 1,000 points, with 900 points or more being rated as a tertiary hospital. A tertiary hospital is the highest level of hospitals in China, based on the classification of hospitals, and it operates with the financial support of the government.) Surgery mainly included cardiothoracic surgery, neurosurgery, hepatobiliary surgery, urology surgery, anorectal surgery, burn surgery, breast surgery, pediatric surgery, plastic surgery, orthopedics and hand surgery, which amounted to a total of 11 departments, and each department employed an average of 10–15 nurses. After communicating with each hospital before the survey and obtaining consent, this study obtained the numbers and information of surgical nurses from each hospital before the physical examination, and all surgical nurses were numbered (starting from 1). The numbers were input into SPSS software, the required samples were extracted using random sampling, and the research objects in the corresponding list were determined. The inclusion criteria for the study subjects were as follows: (1) 18–60 years old; (2) employed at the hospital for at least six months; (3) clinical surgical nurses who held a nurse qualification certificate and who were registered on duty; (4) informed consent provided by nurses and voluntary participation in this study. The exclusion criteria for the study subjects were as follows: (1) nurses who took sick leave or maternity leave during the study period; (2) nurses who were studying and practicing at the hospital; (3) nurses who were not willing to participate in this survey. A total of 550 questionnaires were distributed, and 532 questionnaires were retrieved. Ultimately, 488 valid questionnaires were retrieved, with an effective recovery rate of 88.73 %. The research design was approved by the ethics committee of Xinjiang Medical University. After the questionnaire was issued by the researchers, the nurses completed it by themselves. All respondents provided their voluntary written informed consent before the investigation.

### Research methods

A questionnaire (detailed below) was used to investigate the status of occupational stress, job burnout and quality of life.

### General investigation

This section discusses general demographic characteristics such as sex, age, working years, educational level, marital status, professional title, night-shift frequency, smoking and alcohol use.

### Occupational stress investigation

The Effort-Reward Imbalance questionnaire (ERI) was used to evaluate the level of occupational stress among the participants in this study. This questionnaire was formulated by Johannes Siegrist in 1996 [32]. It was developed under the model of imbalance between pay and remuneration, and consisted of three parts—effort, reward (including salary, respect, career stability and promotion prospect) and internal input—with a total of 23 items. The Chinese version of the ERI scale was introduced by the Yang Wenjie and Li Jian in 2004 [33]. The reliability and validity of the Chinese version of the ERI scale were tested with staff from Zhengzhou Hospital in China, as a sample cohort. After data analysis, the results showed that the alpha coefficient of the effort scale was 0.78, the alpha coefficient of the reward scale was 0.81, and the alpha coefficient of the internal input scale was 0.74, which showed good reliability [34]. Xiuyang et al. [35] argued that the Chinese version of the ERI scale has better reliability and validity in China. The first six items in the ERI scale measure “effort”, the middle 11 items measure “reward”, and the last six items measure “internal input”. The calculation formula of the ERI ratio was as follows: The score for “effort” / (score for “reward” × C), where C is the ratio of the number of “effort” items to the number of “reward” items, i.e., 6/11. If the ERI ratio was > 1, it was regarded as the winner with high effort and low reward (i.e., high occupational stress); if the ERI ratio was ≤ l, it was the winner without high effort and low reward (i.e., low occupational stress) [36].

### Job burnout investigation

The Chinese Maslach Burnout Inventory General Survey (MBI-GS) was used to measure the burnout level among the respondents in this study. The MBI-GS was revised by Maslach and Jackson in 1996 on the basis of the original MBI scale[37]. The Chinese version of the MBI-GS was translated and revised by domestic scholars, Li Yongxin et al., according to Chinese language and culture [38]. This study adopted the scale revised by Professor Li Fuye [39] on this basis, the study showed that the reliability, validity and other measurement indicators of the scale were good and they satisfied the requirements of psychological measurement. The Chinese version of the MBI-GS includes three dimensions, namely, “emotional exhaustion”, “depersonalization” and “lower personal satisfaction”, and it consists of a total of 15 items, i.e., five items for “emotional exhaustion”, four items for “depersonalization” and six items for “lower personal satisfaction”. The questionnaire was scored according to seven levels ranging from 1 to 7, with “1” representing “completely consistent” and “7” representing “completely inconsistent”. The study referred to the grading standard of Ye Zhihong et al. [40] to assess the critical value of job burnout (i.e., exhaustion score ≥ 25, depersonalization score ≥ 11, and lower personal satisfaction score ≥ 16) among nurses. Li Yongxin’s [38] method was used to classify job burnout into four levels: Zero burnout (respondents scored below the critical value with respect to three dimensions of the MBI-BS); mild burnout (respondents scored at or above a critical value with respect to one dimension of the MBI-GS); moderate burnout (the respondents’ scores on two dimensions of the MBI-GS were equal to or above the critical value); and high burnout (the respondents’ scores on three dimensions of the MBI-GS were equal to or above the critical value).

## Quality of life investigation

The Chinese version of the 36-item Short Form Health Survey (SF-36) was adopted [41]. The Chinese version of the SF-36 was translated and revised repeatedly by domestic scholars, such as Li Lu [42], and its performance was tested. The study found that the alpha coefficients of the eight subscales in the Chinese version ranged from 0.78 (general health perceptions) to 0.94 (physical function), reflecting the acceptable internal stability of the Chinese version, which suggests that the Chinese version of the SF-36 had good reliability and validity [43]. The scale included 36 items divided into eight dimensions: physical functioning (PF), role limitations due to physical health (RP), bodily pain (BP), general health perceptions (GH), vitality (VT), social functioning (SF), role limitations due to emotional problems (RE) and mental health (MH). Each dimension included several problems. Among these, the first four dimensions were classified as physiological health, and the four that followed were classified as psychological health. The conversion formula for the score of each dimension was as follows: The conversion score = (actual score - the lowest possible score of this dimension)/(the highest possible score of this dimension - the lowest possible score of this dimension) ×100, the score of each dimension was 0–100 points. The mean of the sum of the scores of the eight dimensions was taken as the total score. The higher the score, the less the level of harm and the better the quality of life [44].

### Structural equation model

Using Analysis of Moment Structures (AMOS) software, ERI, job burnout, and quality of life were set as latent variables, and each index of the three scales was taken as an observation scalar. A structural equation model was established to analyze the path relationship between job stress, job burnout, and quality of life. The steps involved in the AMOS are as follows: establish the model - import relevant data - set the model parameters - evaluate the model - modify the indicators - obtain the best model. In this study, the model fitting indexes were as follows: χ2/ df < 3.0, RMSEA < 0.08, AGFI > 0.9, GFI > 0.9. By using AMOS for analysis, the software disassembled the complex correlation into several linear regression models according to the path diagram drawn by the analyst (path analysis only considers the linear correlation). All of the disassembled linear regression models are generally called structural models, and they are then fitted directly, which can save a lot of time.

### Quality Control

Before the formal investigation, a trial investigation was carried out to further modify and improve the questionnaire, and accumulate experience in the field investigation and organization. The investigators familiarized themselves with the investigation content by conducting a preliminary investigation, and they ensured that the participants were able to complete the questionnaire accurately and thoroughly. Survey implementation stage: The trained investigator distributed the questionnaire to the nurses who participated in the survey. The purpose and content of the study were explained to the participants. The participants completed the questionnaire anonymously, so as to safeguard their privacy and ensure that the questionnaire would only be used for the purposes of this study. Data recovery and entry: The collected questionnaires were reviewed, and questionnaires that failed to comply with the inclusion requirements were excluded. Questionnaires for which > 5 % of items were missing were also excluded. The remaining completed questionnaires were coded and sorted, and the questionnaire results were entered into the database in pairs to ensure the accuracy of the data.

### Statistical methods

SPSS for Windows version 22.0 software (SPSS Inc., Chicago, IL, USA) was used for data processing and statistical analysis. All measurement data used $\stackrel{-}{X}\pm S$ for statistical descriptions. A t-test of two independent samples was carried out to compare the two groups of means. One-way analysis of variance (ANOVA) was used to compare the three groups and the means of more than three groups. Correlation analysis: Pearson’s correlation coefficient was used to analyze the correlation between occupational stress and job burnout among surgical nurses. Multivariate analysis: Multiple linear regression analysis was used to analyze the impact of occupational stress and job burnout on the quality of life of surgical nurses, and the interaction between occupational stress and job burnout on the quality of life. The relationship between occupational stress, job burnout and quality of life of surgical nurses was analyzed using Amos 22.0 software, and the optimal structural equation model was fitted. The significance level was α = 0.05.

## Results

### Quality of life score of surgical nurses under different population characteristics

Male nurses had higher quality of life scores than female nurses (*P* = 0.023); nurses aged 29–28 years old had the lowest quality of life score, and nurses over 39 years old had the highest quality of life score (*P* < 0.001); the quality of life scores of nurses with different professional titles were different (*P* < 0.001); the total score of quality of life of night-shift frequency (> 3 times/month) was lower than that of night-shift frequency ≤ 3 times/month (*P* < 0.001); there was no statistical difference in the total score of the quality of life of nurses in comparison with other population characteristics (*P* > 0.05). Table 1.

**Table 1:** Comparison of quality of life scores according to different population characteristics $\stackrel{-}{\chi }\pm S$

Variables		N	Total score of quality of life	*F*	*P*
Sex	Male	20	73.99 ± 11.94	5.216	0.023
	Female	468	69.14 ± 9.19		
Age group, years	< 29	249	70.47 ± 9.02	14.393	<0.001
	29 ~ 38	195	66.88 ± 9.30		
	> 39	44	73.85 ± 8.77		
Ethnicity	Han	329	69.53 ± 9.42	0.393	0.531
	Minority	159	68.96 ± 9.24		
Working years	< 5	203	69.54 ± 9.59	0.766	0.466
	5–10	189	68.73 ± 9.16		
	>10	96	70.11 ± 9.26		
Educational level	Below bachelor’s degree	345	69.18 ± 9.91	0.324	0.569
	Bachelor degree or above	143	69.72 ± 9.49		
Professional titles	primary	406	68.59 ± 9.42	8.835	< 0.001
	intermediate	72	72.61 ± 7.81		
	advanced	10	76.41 ± 9.76		
Marital status	Single	302	69.19 ± 9.01	0.216	0.642
	Married	186	69.59 ± 9.92		
Night-shift frequency	> 3 times/month	355	68.17 ± 9.34	21.332	< 0.001
	≤ 3 times/month	133	72.47 ± 8.69		
Smoking	Yes	9	71.17 ± 14.97	0.349	0.555
	No	479	69.31 ± 9.24		
Drinking	Yes	33	68.86 ± 9.36	0.095	0.759
	No	455	69.38 ± 9.36		

### Relationship between occupational stress and quality of life

Two independent-samples t-tests were used to analyze the quality of life scores under different stress levels. The results showed that there were 82 ERI ≤ 1 (low-stress group) and 406 ERI > 1 (high-stress group). The total score of the quality of life of nurses in the high-stress group was lower than that observed in the low-stress group (*t* = 2.749, *P* = 0.006). Table 2.

**Table 2:** Comparison of quality of life scores according to different stress levels $\stackrel{-}{\chi }\pm S$

Grouping	N	Total score of quality of life	*t*	*P*
ERI ≤ 1(Low-stress group)	82	71.91 ± 9.47	2.749	0.006
ERI > 1(High-stress group)	406	68.82 ± 9.26		

### The relationship between job burnout and quality of life

Using one-way ANOVA to compare the scores of quality of life according to different levels of burnout, the results showed that, among the four burnout levels, which included zero, low, medium and high, the total score of quality of life was the highest in the zero-burnout group and the lowest in the high-burnout group, and the difference was statistically significant (*P* < 0.001). Table 3.

**Table 3:** Comparison of quality of life scores according to different burnout levels $\stackrel{-}{\chi }\pm S$

Grouping	N	Total score of quality of life	*F*	*P*
Zero-burnout group	27	73.71 ± 8.82	26.767	<0.001
Low-burnout group	117	71.34 ± 9.08		
Medium-burnout group	227	69.74 ± 8.63		
High-burnout group	117	63.73 ± 8.60		

### Correlation analysis of occupational stress and job burnout among surgical nurses

Pearson’s correlation analysis was used to analyze the correlation between occupational stress and job burnout among surgical nurses, and the results showed that there was no statistically significant difference between effort and depersonalization, or between internal input and depersonalization (*P* > 0.05). All other dimensions were correlated with each other (*P* < 0.05 or *P* < 0.01). Among them, lower personal satisfaction was negatively correlated with effort and reward, while the other dimensions were positively correlated. Table 4.

**Table 4:** Pearson’s correlation analysis of occupational stress and job burnout among surgical nurses

Variables	Emotional exhaustion	Depersonalization	Lower personal satisfaction	Job burnout score
Effort	0.522**	0.028	-0.309**	0.391**
Reward	0.354**	0.163**	-0.356**	0.396**
Internal input	0.487**	0.034	0.286**	0.368**

Note: * *P* < 0.05; ** *P* < 0.01

### Effects of the interaction of occupational stress and job burnout on the quality of life of surgical nurses

To further analyze the impact of the interaction between occupational stress and job burnout on quality of life, we analyzed the pairwise interaction of stress and burnout with quality of life using multiple stepwise linear regression. The results showed that the interaction between stress and burnout had an effect on quality of life (*P* < 0.001), and high stress coupled with high burnout had the greatest effect on quality of life Table 5.

**Table 5:** Correlation analysis between occupational stress and job burnout among surgical nurses and their quality of life

Variables	*β*	*S.E.*	*β’*	*t*	*P*
Constant	72.797	0.648	-	112.364	<0.001
Low stress * Low burnout	-8.289	2.977	-0.119	-2.785	<0.001
High stress * Medium burnout	-3.296	0.905	-0.172	-3.604	<0.001
High stress * High burnout	-9.133	1.060	-0.406	-8.618	<0.001

Note: * representational interaction

### The structural equation model of occupational stress - job burnout - quality of life

AMOS 22.0 software was used to conduct the maximum likelihood estimation test, and the following model was selected according to the principles of accuracy and conciseness through continuous modification of the model. Among all of the models, the overall fitting effect of the model was the closest to the reference standard (${\chi }^{2}$/df < 3.0, RMSEA < 0.08, AGFI > 0.9, GFI > 0.9). The model had good fitting (${\chi }^{2}$/df = 1.803, RMSEA = 0.041, AGFI = 0.946, GFI = 0.962) and statistical significance (*P* < 0.001).

**Fig. 1:**
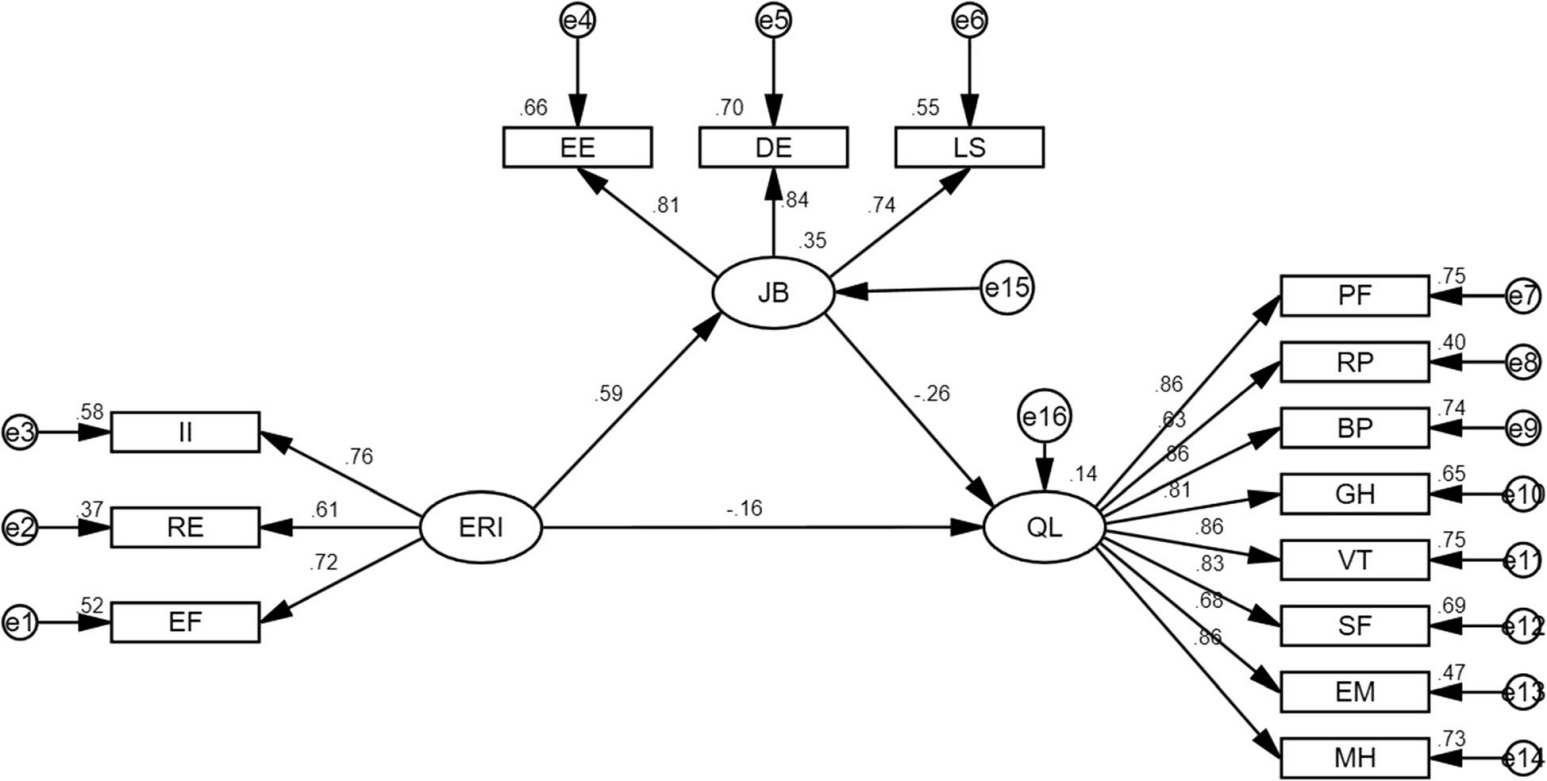
Structural equation model of occupational stress - job burnout - quality of life. Note: II = Internal input; RE = Reward; EF = Effort; JB = Job burnout; EE = Emotional exhaustion; DE = Depersonalization; LS = Lower personal satisfaction; QL = Quality of life; PF = Physical functioning; RP = Role limitations due to physical health; BP = Bodily pain; GH = General health perceptions; VT = Vitality; SF = Social functioning; EM = Role limitations due to emotional problems; MH = Mental health.

As can be seen from the figure: (1) The direct effect of occupational stress (ERI) on job burnout was 0.59 (*P* < 0.001), indicating that the higher the level of occupational stress, the greater the severity of job burnout; (2) the direct effect of occupational stress (ERI) on quality of life was − 0.16 (*P* = 0.026), indicating that the higher the level of occupational stress, the poorer the quality of life; (3) the direct effect of job burnout on quality of life was − 0.26 (*P* < 0.001), indicating that the greater the severity of job burnout, the poorer the quality of life; (4) An indirect effect was observed between occupational stress and quality of life, and job burnout was the mediating variable of the interaction between these factors. The mediating effect was: 0.59 × −0.26 = − 0.153 Fig. 1.

## Discussion

### Factors influencing the quality of life of surgical nurses

This study found that male nurses had higher quality of life scores than female nurses, which may be because men are superior to women in terms of their physical strength and energy, and they can withstand greater pressure. When confronted with the heavy demands of nursing work, they can better adapt to high-intensity nursing work than women. Women tend to have more family responsibilities in their daily lives and they take fewer breaks than men, which can lead to higher levels of stress. This is consistent with the research results of Thakre et al. [45]. Nurses between 29 and 38 years old had the lowest quality of life scores. This can be explained by how nurses in this age group are independent in life and have just started their career. Their professional title is lower and their income is lower. They need to take on both family and work responsibilities. The total score of quality of life among nurses over 39 years old was the highest. It may be that, as nursing staff get older, they acquire more professional experience, their income increases, their family life tends to be stable, their children are generally capable of living independently, their daily life does not require as much care and they have a stronger ability to cope with the various risks associated with their work. Nurses in this age group often hold important positions in their work, and they can enjoy a greater feeling of victory and satisfaction, so the total score of quality of life was higher. However, most nurses aged between 18 and 28 years old have short working hours after they graduate, and most of them have not yet lived independently. Although they have heavy workloads, they rely on the help of their families, which diminishes some of the work-related pressure. The higher the title, the higher the total score of quality of life, which is consistent with the research results of Mchurh [46]. Studies have shown that higher positions tend to allow for greater room for development. Such positions are associated with a higher degree of freedom from authority, and various needs are easier to satisfy [47]. Senior nurses have more advanced qualifications, mature skills and rich experience, which enables them to cope with the pressures of their work more easily. As the frequency of night shifts increased, the quality of life score of nurses decreased. This finding may be due to the way in which night shifts affect the body’s normal ability to rest and sleep. Frequent night shifts disrupt the body’s biological rhythm, which means that it is more difficult to recover from fatigue by ensuring sufficient rest, which thus reduces the quality of life [48].

### Relationship between occupational stress job burnout and quality of life among surgical nurses

Among the 488 respondents who participated in this study, 82 nurses exhibited low levels of stress and 406 nurses exhibited high levels of stress. The quality of life score among nurses in the low-stress group was higher. Studies have shown that long-term occupational stress can lead to a decline in occupational quality of life, which may be caused by psychological stress, and it then affects work [49]. Some studies have confirmed that occupational stress among medical staff can not only cause a decline in the individual’s quality of life, but it can also lead to a reduction in the standard of medical services provided. This may in turn contribute to an increase in occupational risk and the loss of medical staff, which generates a serious economic burden for society [50]. Compared with the zero, low, medium and high burnout groups, the quality of life score decreased as the severity of burnout increased. Scott et al. [51]. argued that job burnout can seriously affect one’s physical and mental health, work, and organization. Job burnout is closely related to work efficiency. The higher the severity of job burnout, the lower the level of work efficiency, and the greater the likelihood of absenteeism and job-hopping. Job burnout is one of the possible occupational health problems observed among nurses. An increasing number of studies have proved that nursing is associated with a high incidence of job burnout [52, 53]. There is a correlation between each dimension of occupational stress and each dimension of job burnout, which indicates that stress and burnout are also mutually interactive. Peihong et al. [54] proposed that occupational stress and burnout interact with each other, causing serious harm to the physiology and psychology of nurses. If an individual cannot effectively cope with occupational stress in the workplace, long-term burnout will ensue, leading to higher stress levels [55].

By carrying out a multiple stepwise linear regression analysis of the factors affecting quality of life, it was found that, in addition to the characteristics of age, professional titles and frequency of night shifts, the quality of life of nurses was also affected by internal input, the ERI ratio, lower personal satisfaction and job burnout. The interaction between stress levels and the burnout level was further analyzed to determine the impact on quality of life. The results showed that the interaction between occupational stress and job burnout affects quality of life. SEM can deal with multiple variables at the same time and estimate factor structure and factor relationship, which has been widely used in current research. The study found that [56], through the establishment of a structural equation model, job satisfaction of rural primary health care workers in China has a direct impact on job burnout and resignation intention, while job burnout has a direct impact on resignation intention, and job burnout is the mediating variable. Job demands of social workers have a significant positive impact on their job burnout, but have no significant impact on their job engagement [57]. SEM analysis of the stigmatization of psychologists found that, psychological flexibility was found to predict stigma indirectly via burnout [58]. All these studies have proved that SEM can reflect the effect relationship between variables well. This study also confirmed the internal relationship among the variables through the establishment of SEM. The results show that, both occupational stress and job burnout had a strong impact on quality of life. At the same time, job burnout has a mediating effect on occupational stress and quality of life. Hu Huihui [59], a domestic scholar, carried out an analysis of the professional quality of life of general surgery nurses, and the results revealed an imbalance between effort and return, which was regarded as the most critical factor affecting their professional quality of life. There is a correlation between occupational stress and job burnout and employee health, among which emotional exhaustion has a particularly significant impact on physical and mental health. Job burnout also has a negative impact on an individual’s physical and mental health, and different occupational stress factors may lead to different health problems, and a reduced quality of life [60, 61].

### Suggestions for improving the quality of life of surgical nurses

Therefore, it is necessary to alleviate occupational stress and job burnout among nursing staff, and to increase their job satisfaction, so as to improve the quality of life of those who work in the medical profession. Based on the above analysis, the following suggestions are proposed: (1) Health education should be strengthened to make employees fully aware of the impact of occupational stress and job burnout on quality of life and medical services. It is also important to enhance coping resources and improve the ability of nurses to adjust so that they can alleviate occupational stress and job burnout. Effective health education activities can encourage greater awareness among nurses of the importance of self-care. Such activities can establish good principles of doing things, and help nurses to deal with things rationally. (2) Psychological education and psychological counseling activities should be actively carried out, and professional mental health care and psychological treatment should be provided for nursing staff, so that they can maintain a good psychological state at work. (3) Organization and management should be improved, and occupational stress and burnout should be reduced. (4) A good micro-social psychological environment should be established with various recreational and sports activities, to enhance personal comprehensive cultivation and social support, and to improve the ability to deal with tension and stress. (5) The study of occupational stress and job burnout among nursing staff should be encouraged, to identify high-risk groups and specific influencing factors, and to develop targeted prevention and control measures.

## Conclusions

The overall quality of life evaluation score of surgical nurses was not high, and the findings were not only affected by demographic characteristics, but were closely related to occupational stress and burnout. Therefore, an objective evaluation of the levels of stress and burnout among nurses can be used as a predictor of their quality of life status. A corresponding system should be established according to individual characteristics and stress influencing factors, in order to facilitate active interventions and reduce occupational stress among nurses. Limitations of this study: (1) This study carried out a cross-sectional survey and cannot infer causality. The results of this study should be further verified by high-level studies, such as cohort studies, to demonstrate a causal relationship. (2) The participants in this study only included surgical nurses. In the future, the sample size should be increased and nurses from all departments should be investigated to minimize deviations and verify the conclusions of this study. (3) Due to the limitation of conditions, this study only selected tertiary hospitals in Urumqi, and did not carry out an investigation of hospitals at other levels. It is hoped that hospitals at different levels will be included in future studies.

## Data Availability

The datasets used and analyzed during the current study are available from the corresponding author on reasonable request.
